# Crosstalk between CAFs and tumour cells in head and neck cancer

**DOI:** 10.1038/s41420-024-02053-9

**Published:** 2024-06-26

**Authors:** Xinyang Li, Celia González-Maroto, Mahvash Tavassoli

**Affiliations:** 1 Head and Neck Oncology Group, Centre for Host Microbiome Interaction, King’s College London, Hodgkin Building, London, SE1 1UL UK; 2 Blizard Institute, Barts and the London School of Medicine and Dentistry, Queen Mary University of London, London, UK

**Keywords:** Oral cancer, Cancer microenvironment, Tumour virus infections, Cell signalling

## Abstract

Head and neck squamous cell carcinomas (HNSCCs) are amongst the most aggressive, complex, and heterogeneous malignancies. The standard of care treatments for HNC patients include surgery, radiotherapy, chemotherapy, or their combination. However, around 50% do not benefit while suffering severe toxic side effects, costing the individuals and society. Decades have been spent to improve HNSCC treatment outcomes with only limited success. Much of the research in HNSCC treatment has focused on understanding the genetics of the HNSCC malignant cells, but it has become clear that tumour microenvironment (TME) plays an important role in the progression as well as treatment response in HNSCC. Understanding the crosstalk between cancer cells and TME is crucial for inhibiting progression and treatment resistance. Cancer-associated fibroblasts (CAFs), the predominant component of stroma in HNSCC, serve as the primary source of extra-cellular matrix (ECM) and various pro-tumoral composites in TME. The activation of CAFs in HNSCC is primarily driven by cancer cell-secreted molecules, which in turn induce phenotypic changes, elevated secretive status, and altered ECM production profile. Concurrently, CAFs play a pivotal role in modulating the cell cycle, stemness, epithelial-mesenchymal transition (EMT), and resistance to targeted and chemoradiotherapy in HNSCC cells. This modulation occurs through interactions with secreted molecules or direct contact with the ECM or CAF. Co-culture and 3D models of tumour cells and other TME cell types allows to mimic the HNSCC tumour milieu and enable modulating tumour hypoxia and reprograming cancer stem cells (CSC). This review aims to provide an update on the development of HNSCC tumour models comprising CAFs to obtain better understanding of the interaction between CAFs and tumour cells, and for providing preclinical testing platforms of current and combination with emerging therapeutics.

## Facts

- CAFs can constitute a large component of the TME in late stages of HNSCC. Recent advancements in single-cell and multiomics data analysis highlight the crucial role of the TME components including CAFs in HNSCC treatment failure.
- In HNSCC, the activation of CAFs is primarily initiated by tumour cells through the secretion of molecules, inducing changes in the phenotype and secretory profile of CAFs. CAFs and tumour cells interaction mediates a pro-tumoral effect on cancer cells, resulting in faster proliferation, increased stemness, EMT, and therapeutic resistance.
- CAFs have also been shown to elicit tumour suppressive effects through paracrine signalling. CAF-tumour cell bidirectional interactions primarily mediate a pro-tumoral effect. Simultaneously, CAFs have also been reported to elicit tumour suppressive effects through paracrine signalling.
- Relevant 3D in vitro cancer models present a cost-effective platform for preclinical testing and the study of TME/cancer cell interactions.

## Open questions

- Currently, there is no standardized method to differentiate CAFs from normal fibroblasts (NFs). What is the cause of the heterogeneity in the phenotype and function of CAFs in HNSCC and its impact on HNSCC treatment response?
- Can 3D in vitro models bridge the gap between cell cultures and the disease in vivo? What strategies can be employed to create representative and high throughput cancer models that closely mimic the TME and how this approach might serve as a reliable platform for predicting patient responses to treatment?
- What key molecules, pathways, and effects are mediated by the crosstalk between CAFs and tumour cells in HNSCC, with the potential to develop more effective personalised HNSCC treatment.

## Introduction

**Fig. 1:**
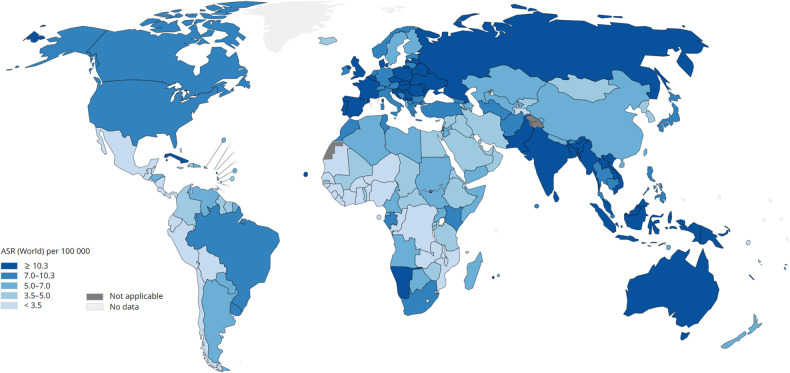
Global incidence of HNSCC. The estimated age-standardized rates (ASRs) of head and neck squamous cell carcinoma (HNSCC) incidence worldwide are shown for men and women combined. The data are from GLOBOCAN for 2020 [1]. The map was generated using the GLOBOCAN website mapping tool by selecting the ‘hypopharynx’, ‘larynx’, ‘lip, oral cavity’, ‘nasopharynx’ and ‘oropharynx’ cancer sites.

Head and neck squamous cell carcinomas (HNSCCs) are a group of malignancies that develop from the mucosal epithelium lining of oral cavity, pharynx and larynx [1]. Currently, HNSCCs ranks the 6th most common malignancy worldwide, with 878,348 new cases and 444 347 deaths in 2020 [2, 3]. The incidence of HNSCC has risen over the past decade and is anticipated to rise further by ~42%, to around 1.25 million new cases annually by 2040 (GLOBOCAN) (Fig. 1). The incidence of HNSCC varies across countries and regions because of strong correlation with different types of risk factors. Classically, main risk factors for all HNSCCs are tobacco and alcohol, while the past few decades have seen a surge in cases of human papillomavirus (HPV)-related oropharyngeal squamous cell carcinoma (OPSCC) caused by high-risk HPV infection (mostly HPV-16, but also -18, -31, -33 and -35) [1, 4, 5].

HNSCCs developed in the oral cavity are generally treated with surgical resection, followed by radiation and/or chemotherapy (known as chemoradiation or CRT) depending on the disease stage. Pharynx and larynx cancers, on the other hand, are usually treated by CRT primarily [6]. Clinically, HPV+ and HPV– HNSCCs are staged differently, as patients with HPV-related HNSCC have more favourable prognosis compared to patients with HPV-negative disease [1]. HPV+ cases are in general more sensitive to radiotherapy (RT) and chemotherapy (CT) [7], therefore, multiple clinical trials aim on RT de-escalation in HPV + HNSCC patients to reduce adverse side effects [8, 9].

Apart from standard of care therapies, targeted therapy Cetuximab [10, 11] and immunotherapy against PD-1 and PD-L1 [12, 13] were first approved to treat advanced or recurrent HNSCC patients in 2006 and 2016, respectively. Nimorazole, a hypoxic radiosensitiser, has also been approved for HNSCC patients in Denmark, however, its therapeutic benefit remains uncertain [14, 15]. Ongoing clinical trials for HNSCC include HMBD-001 (antibody against HER3) [16], Lenvatinib (antibody against multiple receptors of tyrosine kinases) [17], therapeutic vaccines against HPV-related antigens [18, 19], adoptive cell therapy [20] and other immunotherapies [21]. However, despite great efforts to improve treatment efficacy, five-year survival rates remain around 61%, 49%, 41 and 25% for laryngeal, oral cavity, oropharyngeal and hypopharyngeal carcinoma, respectively, measured since 2010 in Europe [1, 22].

Majority of deaths from HNSCCs are caused by local recurrence and distant metastasis [3, 23], which usually follow therapy resistance [24, 25]. It is considered that response failure to CRT and other systematic treatments are closely related to a high heterogeneity and complex HNSCC TME [26, 27]. Non-malignant cells within TME, especially CAFs are crucial determinants of tumour initiation, progression, and therapy resistance [28]. Advances in the next generation sequencing (NGS) together with mass spectrometry-based proteomics has brought new insights into understanding TME of HNSCC, which has shown to have high inter- and intra-tumoural heterogeneity [29]. Due to the varying molecular and functional characteristics of CAFs and crosstalk with cancer cells promoting tumourigenesis, it is imperative for therapeutic strategies to exploit the specificity and diversity of CAFs to enhance targeted therapy effectiveness. A better understanding of CAFs nature is essential for the development new therapeutic strategies. In this review we summarize the key research advancements, CAF heterogeneity and the role in signalling pathways linked to cancer cells in HNSCC.

## Origin and heterogeneity of CAFs

CAFs located in the tumour stroma appear as large spindle-shaped mesenchymal cells with stress fibres [30]. Typically, they are derived from activated local fibroblasts, bone marrow fibrocytes, mesenchymal stem cells and stellate cells [28, 30]. It is well documented that under long-term stress and stimuli from TME, quiescent fibroblasts are irreversibly activated to CAFs leading to enhanced ECM production and cytokine secretion [31, 32]. Alternatively, CAFs emerge as the product of mesenchymal transition of endothelial and epithelial cells or the transdifferentiation of smooth muscle cells, adipocytes and pericytes. CAFs are highly heterogenous based on function, origin, stimuli and molecular signature thus there are currently no specific markers to identify and classify subtypes [33].

Historically, CAFs have been identified and characterized mainly by α-SMA expression [30]. Most research is focused on myofibroblast CAFs (myCAFs) expressing high α-SMA, partially due to 2D cell culture limitations [34–36]. However, this marker has proved to be non-specific and hence CAFs remain poorly characterised. The emergence of single-cell RNA sequencing improved the understanding of CAF heterogeneity in HNSCC [35]. Puram et al. used scRNA-seq to characterise HNSCC TME and identified two automatically clustered fibroblasts apart from resting fibroblasts, namely myCAFs and activated CAF. The myCAFs subset expressed higher level of classical CAFs marker α-SMA, while the second group expressed higher receptors, ligands, and ECM genes, including fibroblast activation protein (FAP) and podoplanin (PDPN) [37]. Similarly, α-SMA expression [38] and TGF-β secretion pattern [39, 40] were used as characterisation markers for CAFs according to multiple HNSCC research studies [37, 38, 40, 41]. Overall, these studies suggested the main functions of CAFs in HNSCCs to include inducing or maintaining cancer cell stemness, activating EMT, promoting proliferation, invasion, and immune modulation (Table 1).

**Table 1:** CAF heterogeneity and phenotype in HNSCC and oral sqaumous cell carcinoma (OSCC).

Tissue	Markers	Function	Reference
OSCC	KGF	High motility	[40]
	TGF-β1	Low motility	
	HGF, MMP3	Reduced tumour incidence	
	α-SMA, CD68	Immune modulation Low CD68+ CAFs predictive of poor outcome	[163]
	Vimentin	Promote EMT, poor outcome	[32]
	α-SMA high CD44, CD90, BMP4 low	Decreased tumour proliferation Increased cancer cell stemness	[38]
	α-SMA low, CD44, CD90, BMP4 high	Increased tumour proliferation Decreased cancer stemness	
	α-SMA high	Senescent CAFs	[154]
	PDGFR-β	Stroma activation	[215]
	Caveolin-1	Promotes CAF activation Inhibits TGF-β/SMAD signalling	
HNSCC	CTHRC1 COL1A1, POSTN, TPM4, MFAP2, PDPN	Pro-metastasis	[37]
	CFD, APOD, CXCL12, GPC3, SEPP1		
	Negative for myofibroblasts and CAF markers	Quiescent fibroblasts	
	ACTA2, MYLK, MYL9	Myofibroblasts	
	α-SMA high	CAF activation, poor outcome	[32, 154]
	Seryglycin	Promote tumour proliferation	[154, 215]
	FAP	Poor outcome	
	PDGFR-α	Chemotaxis	[215, 216]
	POSTN	Tumour stemness, poor outcome	[215]

*α-SMA* alpha-smooth muscle actin, *BMP4* bone morphogenetic protein 4, *PDGFR-β* platelet-derived growth factor beta, *CTHRC1* collagen triple helix repeat-containing protein 1, *COL1A1* collagen type 1 alpha 1, *POSTN* periostin, *TPM4* tropomyosin, *MFAP2* microfibril associated protein 2, *PDPN* podoplanin, *CFD* complement factor D, *APOD* apolipoprotein D, *CXCL12* C-X-C motif chemokine 12, *GPC3* glypican 3, *SEPP1* selenoprotein P, *ACTA2* actin alpha 2, *MYLK* myosin light chain kinase, *MYL9* myosin light chain 9, *FAP* fibroblast activation protein, *PDGFR-α* platelet-derived growth factor alpha.

Extensive research suggests intratumoural heterogeneity amongst malignant and non-malignant TME cells and their interactions play key determinant roles in tumourigenesis. In HNSCC CAFs can account for 80% of tumour mass in late-stage cases [42] and high myCAFs are correlated to poor survival outcomes [35, 43]. It is evident that CAFs are a significant constituent of the TME in HNSCC and influence tumour progression and treatment resistance [44]. Further research is required to better understand the diverse function and subtypes of CAFs and to improve the ability to identify, isolate and target CAFs for therapeutic purposes [31].

## 3D co-culture models: novel in vitro approach of studying TME interactions

**Fig. 2:**
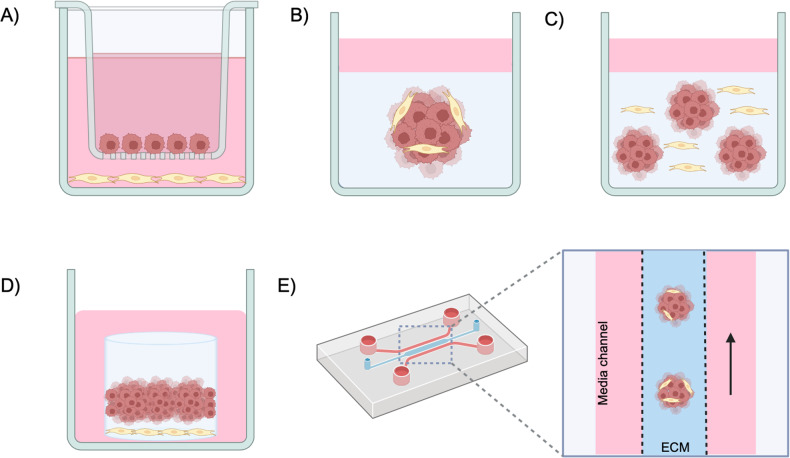
Co-culture models for HNSCC. An illustration of some 2D and 3D co-culture models. **A** Transwell co-culture system. Both HNSCC cancer cells and fibroblasts were seeded and cultured in single layer on top of semi-permeable membrane and bottom chamber, respectively. **B** Spheroid co-culture system. Both HNSCC cells and fibroblasts were pre-cultured in a low attachment dish to form a spheroid, which was then transferred and embedded to collagen or other types of gel matrix. Media was added on top of solidified gel. **C** Organoids co-culture system. Both HNSCC cells and fibroblasts were embedded into gel directly and allowed to self assemble. Media was added on top of solidified gel. **D** Organotypic co-culture system. A feeding layer, composed of either primary NF or CAF, or immortalized fibroblasts and fibroblast derived ECM, served as a scaffold for HNSCC cancer cells. Primary cancer cells or cells lines were then seeded on top of the feeding layer and then lifted to air-fluid interface to induce epithelial polarization and stratification. **E** Microfluidic co-culture models. Microfluidic devices comprise the interconnection of channels which allow simultaneous irrigation of multiple cancer cell populations with constant culture media, which best mimic the shear stress and molecule exchange as in vivo. Cell adhesion and invasion can be easily observed in all the above co-culture models. Pink cells, HNSCC cells. Yellow cells, fibroblasts.

To decipher the diverse functions of CAFs and their intricate interactions with cancer cells it is crucial to replicate the TME architecture. 2D co-culture systems are highly reproducible and cost-effective [33, 42]. However, they fail to mimic the complex tumour structure and kinetics, thus rendered limited and inadequate for studying the complex interactions between TME and cancer cells [45–47]. 3D co-culture models allow in vitro growth patterns that better mimic that of the tissue structure and have become a promising framework for evaluating anticancer treatments [33, 42]. In this section we summarize the main in vitro co-culture systems utilized and their application in HNSCC research (Fig. 2). The main co-culture models can be divided into direct or indirect co-culture models, and 2D or 3D co-culture models. Indirect co-culture is frequently used to study the impact of secreted molecules from one cell type to another cell type. Traditionally this has been achieved by the addition of conditioned media or the use of Transwell system separation in 2D cell cultures. Conditioned media has been successfully employed to investigate the indirect interactions between NF and CAFs with HNSCC cells and has identified several factors such as IL-6, IL8, CXCL-1 [48], MFAP5 [49], TGF-β [50] and IL-1ß [51] as potential therapeutic targets. In HNSCC Transwell semi-permeable membrane system has been widely used to study CAF and cancer cell crosstalk [52–55]. More recently, microfluidic platforms have been utilized for indirect co-culture; as they allow the compartmentalization of heterogenous cell populations in 3D matrices within different microchannels yet allowing media exchange [56].

**Fig. 3:**
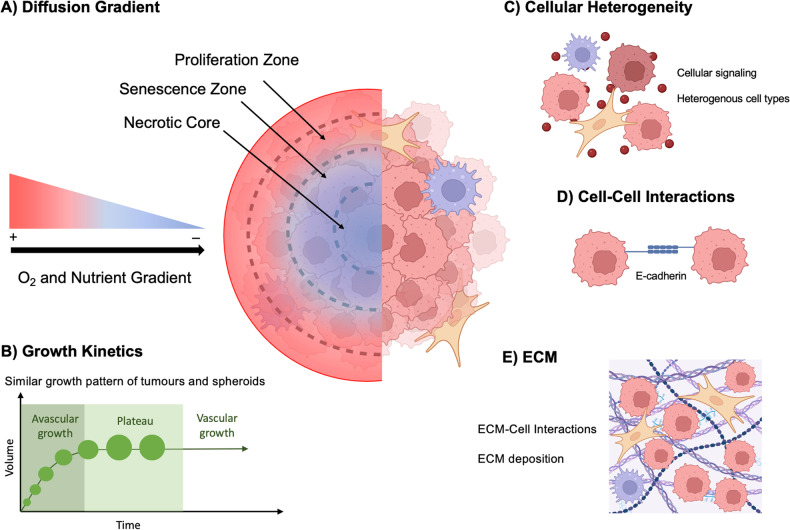
Schematic of spheroid culture characteristics. **A** Hypoxic and nutrient gradients within spheroid cultures including an outer high-oxygen and nutrient region or proliferation zone, a middle medium oxygen/nutrient region or senescent zone followed by a low oxygen/nutrient region or necrotic core. **B** Growth Kinetics of solid tumours (green line) vs spheroids (green circles). Growth profile of tumours and spheroids match presenting an initial exponential growth phase or avascular growth in tumours, followed by a plateau and lastly a invasive phase accompanied with formation of new vessels in vivo. **C** Cellular heterogeneity of cancer cells and stromal cells in spheroid co-cultures can recapitulate cellular distribution and variety present in tumours in vivo. **D** Cell-to-cell interactions are present in spheroid models via cell aggregation and E-cadherin binding of cancer cells. **E** ECM-cell interactions established by ECM synthesis and use of an 3D scaffold of choice.

Direct co-culture methods allow for the physical interaction of different cell types in vitro [45]. More physiologically and clinically relevant in vitro cancer models incorporating TME features, such as 3D tumour geometry, oxygen levels and heterogeneous cell populations, have been integrated with the advances in spheroid/organoid and microfluidic technology bioengineering [47]. However, few tissue-specific TME key features have been applied to in vitro HNSCC models in comparison to other cancer types. Representative HNSCC in vitro tumour models comply with a minimum of one of the following characteristics: (I) Present a 3D tumour-like geometry for ECM-cell and cell-to-cell interactions; (II) Contain heterogeneous cell types combining cancerous and stroma cells and lastly (III) Mimic the hypoxic oxygen level condition present in vivo due to aberrant tumour vasculature. Spheroids can encompass all these characteristics while also replicating tumour growth kinetics and drug diffusion patterns present in HNSCC solid tumours (Fig. 3) [45, 47].

Spheroids and organoids are clusters of cellular aggregates formed in low adhesion culture conditions. However, organoids are composed of patient-derived tissue and are commonly generated from pluripotent stem cells [57–59]. Both present a suitable in vitro model for oncology drug testing [45, 46]. Melissaridou et al. showed increased resistance to cisplatin and cetuximab treatments as well as upregulated EMT-associated stem markers in patient-derived HNSCC spheroids compared to 2D cultures [46]. Moreover, co-culture organoid models have been employed in HNSCC to study the interactions between CAFs and cancer cells. CAFs were shown to promote HNSCC cell proliferation and a mesenchymal phenotype using 3D patient matched derived spheroids in suspension [60, 61]. Spheroids present a more comparable gene expression pattern to that observed in solid tumours in vivo. Wiechec et al. demonstrated a higher number of differentially expressed genes in 3D tumour-CAFs vs monoculture spheroids in comparison to 2D experiments. Interestingly, upregulated genes MMP9 and FMOD in 3D co-cultures were correlated to overall survival of HNSCC patients [60, 61]. In addition, Puram et al. reported a loss of characteristic markers and ligand expression in CAFs derived from primary tumours when cultured in 2D [37]. Altogether, this highlights the importance of generating representative 3D in vitro models for understanding the signalling pathways involved in CAF-cancer cell interactions and the diverse CAF subtypes [42, 45, 47, 60, 61]. Interaction with other components of the TME are known to influence tumour progression. 3D models can be engineered to include ECM components [45, 47]. Spheroids are often embedded in a scaffold of choice that mimics the ECM [62]. In HNSCC the materials to create a 3D biological scaffold include patient-derived decellularized ECM or synthetic polymers as ECM substitutes. 3D scaffold-based in vitro models or organotypic models aim to mimic the tissue of origin TME allowing for cell–cell and cell–ECM interactions [47, 62].

Tumour hypoxia is a common feature of solid tumours such as HNSCC presenting a median oxygen level of 1.3% [63]. Hypoxia is considered a key cause of treatment failure and a negative prognostic factor in HNSCC [64, 65]. Considering its importance it is vital to replicate hypoxic conditions and irregular irrigation for the generation of effective and representative HNSCC cancer models. Hypoxic gradients have been created utilizing 3D in vitro cell culture geometry within spheroids or within microfluidic chambers. OSCC spheroid model developed by Essid et al. displayed higher mRNA expression of vimentin, N-cadherin and carbonic anhydrase within the spheroid hypoxic core [66]. Furthermore, comparison of 2D and 3D spheroid cultures by Basheer et al. demonstrated a high expression of chemokine receptor CCR7 expression in hypoxic spheroids, absent in 2D hypoxic monocultures [67]. Regarding the irrigation of tumours, microfluidic devices present a promising platform to mimic the irregular blood supply by modulating the flow rate of media in the microchannels. Microfluidic technology presents a cell culturing platform with nutrient and waste removal functions within one structure or microfluidic device. This technology allows the recreation of some features of the complex tissue structure of 3D tumours including stromal cells and blood vessels self- or spatially organized into the microfluidic design. Moreover, low oxygen conditions or oxygen gradients can be engineered into the microfluidic chip device to meet the desirable parameters for a specific cancer model [56].

The first microfluidic HNSCC model was designed by Hatterserley et al., and consisted of a polydimethylsiloxane microfluidic device with a syringe pump. Here HNSCC biopsies were exposed to continuous flow of cisplatin and 5-fluorouracil for 7-days and results indicated a decreased cell viability and proliferation in treated vs control groups. This first microfluidic HNSCC model with constant drug irrigation of patient tumour biopsies represented a significant advancement towards personalized treatment and in vitro drug testing [47, 68]. Another novel 3D tumour system in HNSCC is TRACER platform, optimized to incorporate head and neck primary patient CAFs and tumour cell populations [69]. TRACER system consists of a biocomposite strip of cells within a hydrogel and cellulose scaffold rolled onto a cylindrical core. This system allows cell mapping within the 3D spatial location to investigate direct and indirect cell interactions as well as assessing invasion relative to ECM density [42, 70]. Furthermore, the impact of hypoxia on stroma and cancer cells has been explored with TRACER system. However, this system present limitations such as short culturing times, incompatibility with real-time imaging and absence of other cellular components and structures of the TME. Development of representative 3D in vitro models is key for understanding of tumour-stroma interactions and reciprocal signalling pathways.

## Main pathways that mediate crosstalk between CAFs and HNSCCs

**Fig. 4:**
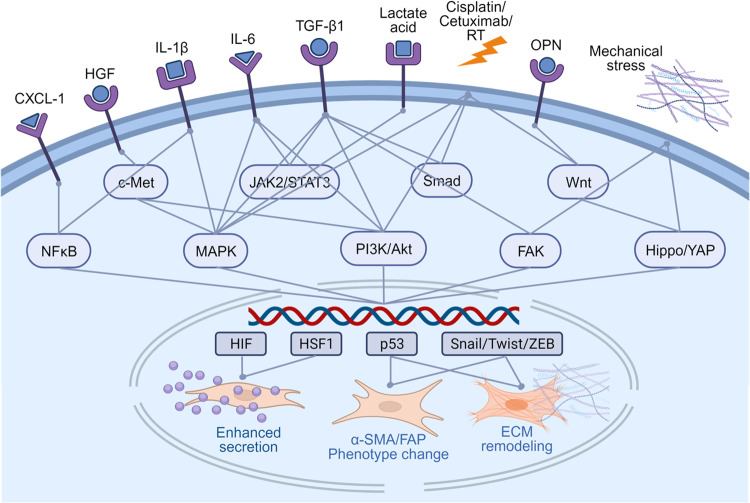
Pathways and mediators associated with CAFs activation in HNSCCs. Activation of fibroblasts in HNSCCs can be triggered by multiple ligands, which can be secreted by either tumour cells or CAFs themselves. The transformation to a more activated state is mediated through several pathways, leading to changes on secretion profile, morphology and ECM remodelling ability of fibroblasts. CXCL-1, C-X-C motif ligand 1. HGF, hepatocyte growth factor. IL-1, interleukin 1 beta. IL-6, interleukin 6. TGF-β1, transforming growth factor-beta1. RT, radiotherapy. OPN, osteopontin. NF-κB, nuclear factor kappa-light-chain-enhancer of activated B cells. c-Met, mesenchymal-epithelial transition factor. MAPK, mitogen-activated protein kinases. JAK3/STAT3, Janus kinase 3/signal transducer and activator of transcription 3. PI3K/Akt, phosphoinositide-3-kinase/protein kinase B. Smad, mothers against decapentaplegic homologue. FAK, focal adhesion kinase. Wnt, wingless-related integration site. Hippo/YAP, Salvador-Warts-Hippo/yes-associated protein. HIF, hypoxia-inducible factors. HSF1, heat shock factor 1. Snail/Twist/ZEB, Zinc finger protein SNAI1/twist-related protein 1/Zinc finger E-box binding homeobox 1. α-SMA/FAP, smooth muscle alpha-actin/ fibroblast activation protein-α. ECM, extra cellular matrix.

**Fig. 5:**
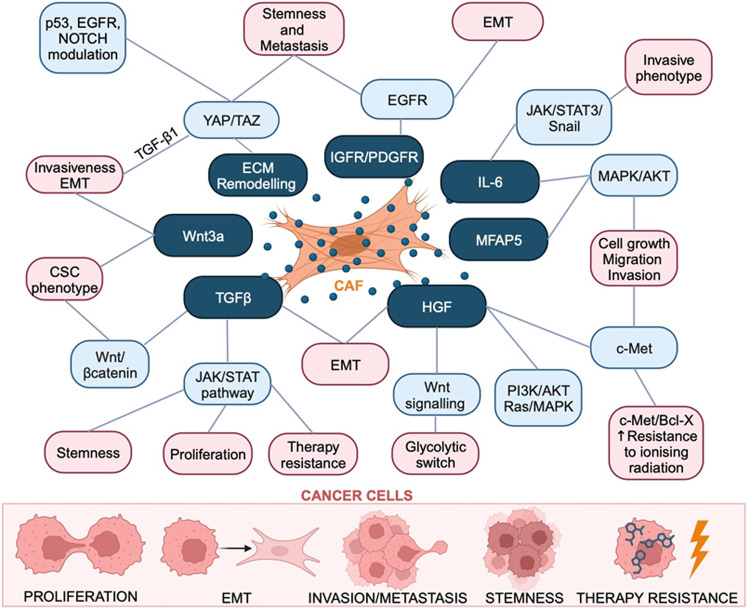
CAF-mediated pro-tumoral effects in HNSCC. CAFs have multiple effects on the progression of HNSCC. Main effects of stromal CAFs on HNSCC tumour cells involve promoting proliferation, induce EMT, enhanced invasiveness and increase the portion of cancer stem cell, and treatment resistance. Response of cancer cells to CAFs is mediated by different molecules and pathways. Some CAFs secreted molecules can activate multiple pathways and changes at the same time in tumour cells. For example, TGF, which is mainly secreted by CAFs in HNSCC, is able to trigger the activation of JAK/STAT, Wnt/βcatenin, Smad, pathways etc., which in turn promote cancer progression in terms of stemness, proliferation and therapeutic resistance in cancer cells. MFAP5, microfibrillar-associated protein 5.

Many signalling pathways have been explored in CAF-mediated induction of cancer progression. The roles that CAFs play in HNSCCs include promoting proliferation, stemness, invasion, migration, EMT, angiogenesis, metabolic modulation, and therapy resistance. Cytokines, chemokines, metabolic products, and miRNAs are all markers involved in the regulation of these signalling pathways. Complicated exchange or interaction of molecules between CAFs and HNSCC cells takes place continually. Therefore, in this section, we will discuss how common signalling pathways mediate crosstalk between CAFs and HNSCC tumour cells in (Figs. 4 and 5).

### TGF-β and related pathway

#### Source of TGF-β in HNSCC TME

TGF-β was first isolated and observed to induce proliferation of rat kidney fibroblast in 1983 [71]. HNSCC tissue usually express higher level of TGF-β [72, 73], which also correlates with more advanced disease and reduced survival [72, 74]. Huang et al. also noticed that a higher level of TGF-β mRNA is correlated with poor prognosis [75]. Contrary, there is evidence to suggest that both the TGF-β receptor, and phospho-fd2 (p-Smad2), one of the main targets of the TGF-β, reduced expression levels, detected by immunostaining were associated with worse prognosis [76, 77].

It is considered that TGF-β is mainly secreted by tumour cells in HNSCC [78, 79], while CAFs also contribute to producing some TGF-β within TME [40, 50]. In HNSCC, TGF-β levels secreted by CAFs are persistently higher compared to those in normal dermal and mucosal fibroblasts [80].

#### Effect of TGF-β on CAFs

TGF-β is initially secreted in its latent form, activated and released in mature status subsequently, and then directly binds to its corresponding receptors on the cell membrane. This interaction initiates a signal transduction cascade that elicits biological actions on almost all cell types [81]. Cell response to TGF-β is achieved by either canonical or non-canonical TGF-β signalling pathways [81]. The canonical TGF-β signalling pathway involves TGF-β/and bone morphogenetic protein (BMP)-Smads pathway. Non-canonical pathway represent those that activate TGF-βR, but induce no-Smads pathway, including MAPK/ERK, p38/JNK and NF-κB, PI3K/AKT/mTOR and JAK-STAT pathways [33, 81].

NFs can be activated by TGF-β through either autocrine or paracrine mechanisms. Oral NFs can be routinely induced to transition into CAFs by TGF-β treatment in vitro [39, 82, 83]. Mechanistically, TGF- β- induced phenotypic transition from NF to CAFs can be triggered by differentially expressed target genes such as α-SMA and FAP in CAFs [84]. At the same time, activated or stressed fibroblasts become secretive, or ‘senescent’ after TGF-β treatment. The secretion pattern of multiple cytokines or chemokines become altered as a result of activation of fibroblasts [38, 50, 83]. Both canonical and non-canonical TGF-β pathways are involved in the activation of NF to CAFs [81, 85].

#### Effect of TGF-β on tumour cells

The CAF-mediated TGF-β pathway contributes to cancer progression by regulating a wide range of biological processes, including cancer cell proliferation or stemness, EMT, metabolism and therapy resistance [32]. It is observed that TGF-β enriches population of cancer stem cells (CSC) in HNSCC, through either Wnt/β-catenin [86] or canonical Smad signalling pathway [87]. However, studies have found a subtype of low α-SMA expressing CAF that suppress oral squamous cell (OSCC) stemness but increased proliferation [38]. TGF-β also serves as a typical trigger of EMT in HNSCC [88]. CAFs have also been shown to mediate ECM remodelling, which provides biochemical and mechanical stimuli for the invasion of cancer cells [89]. Costea et al. found that two subtypes of CAFs, TGF-β-synthesizing fibroblasts (or secretive fibroblasts) and hyaluronan-synthesizing fibroblasts (ECM producing fibroblasts), are both critical for carcinoma invasion as they are responsible for inducing EMT and provide support for tumour cells, respectively [40]. TGF-β-activated CAFs were sufficient to decrease the efficacy of cetuximab, and TGF-β signalling pathway was found to be upregulated in the stromal cells of patient-derived xenografts that derived from patients who did not response to cetuximab treatment [90]. On the other hand, blocking of TGF-β receptor kinase I by galunisertib, was shown to induce a significant radio sensitizing effect on HNSCC cells [91]. Similarly, TGF-β silencing sensitized HNSCC cells to cytotoxic therapies such as cisplatin or paclitaxel [92, 93]. TGF-β also acts as EGFR agonist together with EGF, which triggers the activation of EGFR pathway inducing multiple pro-tumour effects in HNSCC [94].

### MAPK pathway

Nearly 20% of HNSCC harbour mitogen-activated protein kinase (MAPK) pathway mutations, which are largely activating mutations [95]. The MAPK pathway regulates expression of proteins involved in cell proliferation, differentiation, apoptosis, angiogenesis, invasion and metastasis in HNSCC [7]. MAPK signalling pathways comprises signalling cascades involving three major kinases: ERK, c-Jun-N-terminal kinase (JNK), and p38 [96]. MAPKs respond to different types of stimuli, like growth factors (EGF, HGF, FGF), cytokines (TGFβ, IL-6) and environmental stresses (hypoxia, ROS) [96].

#### MAPK pathway in CAFs

TGF-β is an activator of MAPK pathway in multiple fibroblast cell lines [97]. Also, HNSCC cell secreted FGF induces ERK phosphorylation, which in turn regulates HGF production in CAFs. Both FGFR and p-ERK inhibition can block this effect [98]. The JNK/p38 MAPK signalling pathway plays a pivotal role in the formation and activation of CAFs in many cancers, as p38 was proved to maintain CAF phenotype and cytokine secretion pattern in lung cancer and normal dermal fibroblast [99–101].

#### MAPK pathway in tumour cells

CAF-secreted MFAP5 triggers or promotes OSCC cell growth and migration via activation of MAPK and AKT pathways [49]. As typical downstream targets of EGFR pathway, p-AKT and p-MAPK expression were maximally inhibited by targeting both the EGFR and c-Met pathways. This inhibition led to the suppression of proliferation, invasion in vitro and tumour growth in vivo [102]. Another typical activator of the MAPK pathway, IL-6, mainly secreted by CAFs, is also an effective trigger of MAPK activation [103]. IL-6 can induce higher stemness, invasion, and angiogenesis in HNSCC, at least partially via the MAPK pathway [104, 105].

IL-8 treatment induced p-p38 MAPK and p-ERK expression and increased the expressions of p-IκB-α and nuclear factor (NF)-κB, both markers for inflammatory response, through modulating the MAPK and NF-κB pathways in HNSCC cells [106]. In vitro experiments in HNSCC show the induction of ERK and successive VEGF release after irradiation, which might be partially explained by DNA damage repair mediated by the VEGF/ERK pathway [107]. Similarly, the expression of p-ERK1/2 returned to high level after prolonged cetuximab administration and could be induced by fractionated IR. This induction could be suppressed by a MEK inhibitor used as a radiosensitizer [108]. CAFs are one of the main sources of HGF in HNSCC [109], and it is shown that PD-L1, a potential marker of immune checkpoint inhibitor (ICI) treatment failure, is induced upon HGF stimulation in a MAPK-dependent manner [110]. Unexpectedly, MAPK pathway activation were shown to be associated with long HNSCC patient survival, probably via abrogating ErbB3 activation, a well-established progression signal in HNSCC [95].

### EGFR pathway

The epidermal growth factor receptor (EGFR) belongs to the HER/ErbB family of receptor tyrosine kinases (RTKs), which also includes HER2-4. EGFR is overexpressed in 80–90% of HNSCC cases and correlates with poor prognosis and treatment resistance [111]. ErbB family members can be activated by many ligands, including EGF, HGF, heparin-binding EGF-like growth factor and TGF-α [112]. Ligand-dependent activation of EGFR transduces multiple signalling pathways such as PI3K/Akt and Ras/MAPK pathways [113].

#### EGFR pathway in CAFs

EGFR is expressed in almost all neoplastic and nonneoplastic cell types in HNSCC TME, including CAFs [114]. EGFR blocking results in a transition from a NF to CAF phenotype. This transition partially elucidates the role of CAFs in the development of resistance in HNSCC. Expression pattern of CAF markers and related genes, including ACTA2, CXCL12, FAP and TGF-β1 after treatment with cetuximab in HNSCC patients was measured, and an induction of CAF phenotype was noticed [115].

#### EGFR signalling pathway-mediated crosstalk of CAFs with cancer cells

EGFR pathway activation induces a wide range of effects, including differentiation, proliferation and survival [7].

Cancer-related ECM is composed of thick collagen bundles organized by CAFs within TME. HNSCC cell collective invasion is driven by the matrix-dependent mechano-sensitization of EGF signalling in cancer cells [55]. By contrast, suppression of integrin signalling inhibits the invasion of epithelial cells as cell to ECM adhesion favours EGFR-dependent cancer proliferation [116]. Activation of EGFR pathway by EGF treatment induces stronger ability of invasion and migration, and also a higher level of EMT markers in HNSCCs [117].

Modulators of EGFR, insulin-like growth factor receptor (IGFR), and platelet-derived growth factor receptor (PDGFR) activity were identified as paracrine cytokines secreted more by CAFs than NFs [118]. Blocking EGFR signalling effectively inhibited CAF-promoted stemness in HNSCC cells [118]. Increased expression of EGFR was observed in HNSCC cells cultured with CAFs, which correlated with increased positive Ki67 cells. EMT and CSC phenotype were favoured in the presence of CAFs in 3D co-culture models [61]. However, inconsistent response of HNSCC to cisplatin and cetuximab with or without the presence of CAF indicates further investigation is needed in order to understand the crosstalk mechanism between CAF and tumour cells [61, 119].

### Hippo pathway

The Hippo pathway is a highly conserved signalling pathway across higher-order vertebrates that modulates key target genes involved in cellular proliferation, stemness, invasion and therapy resistance in cancer [120]. Central to this signalling is a kinase cascade leading from the tumour suppressor Hippo (Mst1/Mst2) to the oncogenic YAP/TAZ, which is a transcriptional coactivator of target genes involved in cell proliferation and survival [121]. Mutations in the YAP gene [122, 123] and recurrant amplification of YAP gene containing 11q22 [123] occurs in some types of carcinomas, including HNSCC. Functional loss of FAT1, a known upstream suppressor of Hippo signalling, leading to the activation of YAP and TAZ is a frequent event in HNSCC [124].

#### Hippo pathway in CAF

The Hippo pathway is activated by stromal stiffness in solid tumour tissues, and a growing body of evidence suggests that the transcriptional factor YAP is activated in CAFs [125, 126]. The oncogenes YAP/TAZ are suggested as being part of the remodelling processes exerted by CAFs. When the ECM becomes stiff, YAP/TAZ gets transcriptionally active and promote CAF function which further stiffens the ECM [127].

#### Hippo pathway in tumour cells

An association between Hippo/YAP expression and HNSCC nodal metastasis was reported, suggesting an involvement of YAP in metastasis [128]. Also, a subset of malignant cells expressing a partial EMT programme with stronger YAP expression was shown to localize to the invasive front of tumours in proximity to CAFs [37]. In vitro experiments show that TAZ, the downstream effector of Hippo signalling, increases tumour cell stemness, promotes EMT, and was involved in TGF-β1-induced EMT in oral cancer cells [129]. Similarly, in OSCC, YAP1 was also shown to be a strong driver of oncogenesis and metastasis [130].

YAP/TAZ-mediated transcriptional regulation appears to crosstalk with many other oncogenic drivers in HNSCC, including Notch [131], p53 [132], and EGFR [133] pathways. The correlation between amplification of TP63 and YAP1 activity is still debated and needs further validation in HNSCC [134, 135].

### Jak/STAT pathway

JAK/STAT signalling pathway mediates many cellular processes initiated by growth factors, hormones, and cytokines [136]. JAK/STAT is a signal transduction pathway, which causes STATs translocation into the nucleus to target the promoter region of genes to regulate processes including cell proliferation, stemness, malignant transformation and invasion [137].

#### Jak/STAT pathway in CAF

JAK/STAT signalling pathway is constitutively activated in CAFs. In TME, CAF-derived cytokines, including IL-6, IL-8 and CXCL-1, act as ligands for JAK/STAT signal cascade [48, 78, 138], some of which can also be secreted from tumour cells [78].

High Epiregulin expression in CAFs has been linked to poor clinicopathological characteristics and shorter overall survival in OSCC. CAF secretes higher level of Epiregulin, which in turn reprograms CAFs via JAK2-STAT3 pathway [139]. Similarly, it has been reported that JAK-STAT can promote the proliferation and activation of fibroblasts from different sources [140, 141].

#### Jak/STAT pathway in tumour cells

Both HPV+ and HPV− HNSCCs demonstrate aberrant regulation of JAK-STAT signalling, upregulation of STAT3, and altered target gene expression pattern. An activated JAK-STAT pathway contributes to higher cell stemness, proliferation and therapy resistance [142, 143]. The constitutive activation of the JAK/STAT pathway has been found to be triggered by several ligands, including EGF, TGF-β, IL-6, IL-10, and IL-22 in HNSCC, most of which are secreted by CAFs [144, 145].

Jak inhibitor was able to halt proliferation of the HNSCC cell lines and downregulate pSTAT3 in vitro and in vivo [146]. Exogeneous or overexpressed IL-6, a typical ligand for Jak/STAT pathway activation in HNSCC cell lines, induced significantly higher expression of EMT markers via the JAK/STAT3/Snail signalling pathway, and cause a more invasive phenotype and poor prognosis [147]. Elevated circular RNA, FAT1, in HNSCC unifies and regulates the positive association between cancer stemness and immune evasion by promoting STAT3 activation [148]. It is also shown that Jak/STAT3 activation was frequently present in EGFR inhibitor resistance HNSCC cases, and addition of STAT3 inhibitor to EGFR blocking strategies significantly enhanced antitumour effects in vivo [149].

### Wnt pathway

Wnt signalling pathway consists of a canonical (β-catenin dependent) and two non-canonical pathways (Wnt/PCP, Wnt/calcium) [150]. During the activation of canonical Wnt pathway, Wnt ligands mediate the activation and translocation of β-catenin to the nucleus for targeted gene expression, including CD44, c-Myc, and cyclin D1 [151]. Increased Wnt signalling was associated with a more advanced clinical stage in HNSCC. These phenomena suggest a potential key role of Wnt signalling in the cancer-stroma crosstalk [152].

#### Wnt in CAF

Wnt activation occurs via autocrine and/or paracrine signalling [150, 153]. Wnt3a is known to be an “activating ligand” for CAFs in many cancers, including HNSCC. Le et al. demonstrated that Wnt pathway proteins were most abundant at the cancer epithelial-stromal boundary and was linked to a poor prognosis in HNSCC. Specifically, Wnt signalling can be initiated in HNSCC cells which then activate CAFs, and in turn perpetuate an enhanced paracrine signalling loop [152].

#### Wnt in crosstalk between CAFs and tumour cells

CAFs co-cultured with cancer cells resulted in Wnt activation in several HNSCC cells lines. This effect was proved to be mediated by CAF-released Wnt3a and can be blocked by Wnt inhibitors. Wnt pathway activation was shown to be responsible for increased CSC characteristics in tumour cells like sphere formation and invasiveness. [152]. Either the presence of CAF or forced overexpression of Wnt pathway gene induce higher invasiveness, more EMT-like phenotype and higher stemness in HNSCC cells lines [152]. Similarly, Xie et al. showed that SRGN secreted by CAFs in hypoxic TME can activate the Wnt/β-catenin pathway and then signalling pathways related to cell stemness, chemoresistance and accelerated tumour growth in a tongue cancer model [154].

### NOTCH pathway

A 2015 genomic analysis by the Cancer Genome Atlas (TCGA) showed inactivating mutations in NOTCH1-3 to be present in 17% of HPV-positive and 26% of HPV-negative HNSCCs [155]. NOTCH is considered to have a bimodal role as a tumour suppressor and an oncogene in HNSCC [156]. NOTCH gene has 4 receptors (NOTCH1-4), however, the downstream targets and the precise mechanisms of each NOTCH subtype remains to be elucidated [7].

#### NOTCH pathway in CAFs

In OSCC, NOTCH3 expression in CAFs was positively correlated with micro-vessel density in cancer stroma and tumour size. Meanwhile, NOTCH3 expression in normal human dermal fibroblasts can be stimulated by direct co-culture with OSCC cell lines, which serve as a pro-tumour loop [157]. NOTCH1 signalling in CAFs serves as a molecular switch, which modulates tumour heterogeneity and aggressiveness by regulation of the plasticity and stemness of CSCs in melanoma [158]. Loss or downmodulation of the Notch effector CSL in oral fibroblasts is sufficient for CAF activation and promotes progression of keratinocyte-derived tumours. Concomitant loss of CSL and p53 overcomes fibroblast senescence, enhances expression of CAF effectors, and further promotes stromal and cancer cell expansion [159]. Generally, the roles that NOTCH pathway play on fibroblast activation and secretory profile needs further investigation.

#### NOTCH pathway in tumour cells

Most NOTCH1 mutations in patient samples are considered inactivating, indicating that NOTCH1 is a tumour suppressor gene. However, most in vitro experiments show that NOTCH promotes HNSCC progression [156]. NOTCH overexpression has been linked to high HNSCC stage [160], tumour aggressiveness [161] and cisplatin resistance [162]. NOTCH1 inhibition reduces CSCs frequency either alone or in combination with chemotherapeutic agents like cisplatin [163, 164]. Similarly, activated NOTCH2 [165], NOTCH3 [166], NOTCH4 [167] signalling are correlated with more aggressive cancer and poor prognosis. In addition to the crosstalk with CAF, Notch ligand Jagged 1, induced by growth factors in HNSCC cells, triggered Notch activation in neighbouring endothelial cells and promoted angiogenesis [168]. On the other hand, decreased mRNA level of NOTCH1 in HNSCC [169] and *Notch3* gene methylation during tumourigenesis of HNSCC [170] present a controversial understanding of NOTCH pathway in HNSCC.

### MET pathway

Mesenchymal-epithelial transition factor (c-Met) is a tyrosine kinase receptor, usually found to be expressed on the surface of epithelial cells. c-Met can be activated by protein overexpression or paracrine/autocrine signalling of HGF [171]. Met pathway activation is present in more than half of HNSCC cases [172].

It is widely agreed that the only source of HGF in TME of HNSCC is CAFs [109, 173]. Compared to NF, CAFs are also characterized by secreting higher level of HGF [80]. Cell growth and invasion ability of HNSCC cells were stimulated by HGF in a c-Met-dependent manner [173]. HNSCC cells and CAFs have a metabolic relationship where CAFs secrete HGF to induce a glycolytic switch in HNSCC cells, which in turn secrete FGF to promote lactate consumption by CAFs [98]. Cetuximab, the only approved anti-EGFR treatment for HNSCC, is only able to induce a long-lasting response in a low percentage of patients [174]. HGF/Met pathway has been suggested to act as a resistance mechanism against EGFR inhibition in advanced HNSCC and co-targeting both receptors has demonstrated improved ability to sensitize cells to cetuximab [102]. A novel MET mutation (R1004G) was identified in a platinum refractory recurrent OSCC patient, who had rapid response to oral MET tyrosine kinase inhibitor crizotinib [175]. HGF/c-Met and Bcl-xL appear to be involved in the resistance of oropharyngeal cancers to ionizing radiation in vitro and in vivo [176]. The ficlatuzumab (an anti-HGF mAb)-cetuximab dual treatment for pan refractory, recurrent/metastatic met significance criteria for PFS and warrants phase III development [177].

## HPV status

Oropharyngeal SCCs are divided into HPV-negative and HPV-positive diseases, two subtypes of diseases have distinct pathophysiological mechanisms and clinical characteristics [1, 178]. HPV is a risk factor associated with 22% of oropharyngeal (OPSCC) and 47% of tonsillar squamous cell carcinomas (TSCC). The incidence of HPV-positive HNSCC increased by 225% from 1984 to 2004 and has now surpassed the incidence of HPV-negative HNSCC in the UK, although there is a significant global variation [179, 180]. HPV-positive cases present with an almost 60% reduction in the risk of mortality after adjustment for prognostic factors, such as age, ethnicity, staging, smoking status and treatment regime [181]. It has also been postulated that HPV-positive tumours harbour fewer genetic mutations and are more radiosensitive, which is associated with an overall better response to radiotherapy [15, 180]. High-risk human papillomaviruses (HPV), in particular HPV-16, but also -18, -31, -33 and -35 are recognized as independent risk factors for this subset of HNSCC [182–184].

Briefly, high-risk HPVs produce 2 oncoproteins, E6 and E7, which are necessary for viral replication. The HPV-E6 protein binds and promotes the degradation of the tumour suppressor p53, diminishing the ability of the cell to undergo apoptosis. The HPV-E7 protein binds and inhibits the retinoblastoma protein (pRb), preventing it from suppressing the transcription factor E2F, resulting in loss of cell cycle control [185].

HPV-positive HNSCCs display significantly lower levels of chromosomal mutations and loss than HPV-negative tumours [186]. While TP53, CCND1, CDKN2A, FGFR1, PIK3CA, and NOTCH are highly mutated in HPV-negative HNSCC [155], HPV positive HNSCC presents a higher mutational incidence of FGFR2, FGFR3, PIK3CA and KRAS genes [178]. Further comprehensive analysis of TCGA data also established the immunologically active nature of two types of HNSCCs as majority of HPV negative cases are immunologically cold tumours compared to their HPV positive counterparts [187].

Rahrotaban et al. noticed that a higher expression level of p16 in HPV-positive HNSCCs is correlated with a higher histopathologic grade and a more intense expression of α-SMA at the same time. Expression of α-SMA has also been linked to a higher histopathologic grade [188]. Similarly, Wang and colleagues found that fewer CAFs infiltrate in HPV-positive compared to HPV-negative HNSCC, a factor that has been linked to a favourable prognosis [78].

In HPV+ cancers, the HPV life cycle is closely related to the stromal cells around it, although this remains an underexplored area, it is known that fibroblasts support HPV interaction with epithelial cells as well as their infection, which ensures the non-activation of host immune surveillance and disease progression [189]. In HNSCC, fibroblasts were also commonly found to be related to immune microenvironment in HNSCC [27]. CAFs support immune evasion and proinflammatory activities by increasing the expression of proinflammatory genes and growth factors, including CXCL1, COX-2, IL-1β, IL-6, TGF-β, and by recruitment of mast cells, macrophages, and neutrophils. In summary, CAFs are involved in both earlier stages of HPV-related carcinogenesis, and also prolonged stimulus for maintenance of cancer cells including acquire stemness and EMT phenotype [5, 180, 188].

Current published research investigates the differential expression and function of CAFs in HPV+ and HPV– HNSCC and demonstrates significant differences in the interactions between two types of cells. HPV + HNSCC‐derived exosomal miR‐9‐5p inhibits TGF‐β mediated fibroblast activation through NOX4, which is correlated with a better prognosis of HPV + HNSCC patients [78]. Bolt et al. noticed that HPV-negative HNSCC cell lines, but not HPV-positive ones, can induce a rapid fibroblast secretory response in CAFs. These CAFs release higher levels of HGF and IL-6, which support cancer cell migration and invasion in both 2D and 3D models [190]. Similarly, Al-Sahaf et al. observed that the secretion of chemokines by fibroblasts is driven by the interaction between HPV-negative HNSCC cells and stromal fibroblasts through an IL-1/IL-1R-mediated mechanism, which is less prominent within the HPV-positive tumour microenvironment [51].

PD-L1 and PD-1 expression levels are found to be upregulated in HPV-related HNSCCs, which is also indicative of a better response to immune therapy and prognosis [191–193] (8,9,10). However, very few studies focused on the underlying crosstalk among TME components that lead to the phenomenon and are inconclusive [4, 194].

## CAF-targeted therapy

It is evident that CAFs are linked to poor prognosis due to their pro-tumorigenic impact on cancer cells. Moreover, CAFs are considered to be responsible for increased resistance to radiotherapy, chemotherapy, targeted therapy, and immune therapy [25, 90, 115, 176]. Inversely, CAFs may also restrain tumour progression in some cancers or interfere with specific stages of cancer progression and treatment [54, 195, 196]. CAFs have complicated crosstalk with other components of the TME and therefore their modulation needs to be considered in the context of their complexicity and multiple roles in cancer progression and treatment.

Immune therapy emerges as one of the essential treatment in many malignancies at advanced stage. However, only 15–20% of patients with HNSCC achieve a durable response to anti-PD-1 or PD-L1 agents despite a twofold to threefold higher expression of PD-1 and PD-L1 within the tumour [197]. CAF, as the main component in TME, have an intimate interaction with immune cells. CAFs are considered capable of suppressing antitumour immune responses mediated by T cells, tumour-associated macrophages [198], and secreted molecules, including IL-6 and TGF-β in HNSCCs [199].

CAFs are related to patients’ response to anti-PD-1 and PD-L1 treatment in HNSCC, as CAFs themselves express PD-1 and PD-L1 [199, 200]. Specifically, two subsets of CAFs in HNSCC emerged as predictive of nivolumab response and were found to reduce TGF-β-dependent PD-1+/TIM-3+ exhaustion of CD8 T cells and enhance the overall cytolytic profile of T cells [26]. Yann et al. verified the presence of a subset of CAFs in HNSCC, which upregulates PD-1 and CTLA4 protein levels in immunosuppressive regulatory lymphocytes (Tregs) [201]. Elevated levels of the TGF-β reprogramming LRRC15 + CAF signature correlated with a poor response to anti-PD-L1 therapy in six cancer types, including HNSCC [202].

**Fig. 6:**
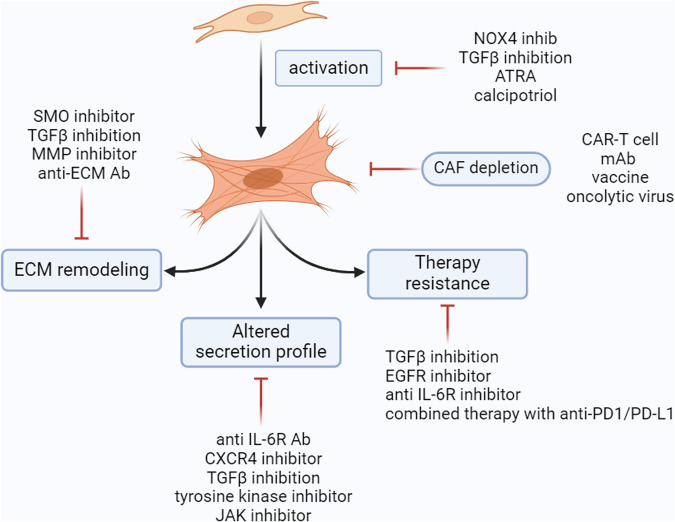
Strategies for CAF-targeted therapy. CAFs can be targeted directly by either CAF reversion or normalization (e.g., NOX4 inhibitors or ATRA) (**a**) or CAFs depletion (e.g., FAP-based depletion by CART-cell or mAb) (**b**). The effect of CAFs produced ECM can be targeted by inhibiting ECM dissolving (e.g., MMP inhibitor) or anti-ECM protein antibodies (**c**). Antibodies against secreted molecules, mainly cytokines and chemokines secreted by CAFs, are potential treatment candidates (e.g., anti-IL-6 and CXCR4 inhibitors). Finally, shared pathways by CAFs and tumour cells are mainly used to tackle CAFs-induced therapy resistance in cancer, including immune therapy by anti-PD-1 and PD-L1 (e.g., TGF pathway inhibitors and TKI inhibitors). NOX4, NADPH oxidase 4. TGFβ, transforming growth factor-beta. ATRA, all-trans retinoic acid. CAR-T, chimeric antigen receptor T-cell. SMO, smoothened. MMP, matrix metallopeptidase. mAb, monoclonal antibody. ECM, extra-cellular matrix. IL-6R, interleukin-6 receptor. CXCR4, C-X-C chemokine receptor type 4. JAK, Janus kinase. EGFR, epidermal growth factor receptor. PD-1, programmed cell death 1. PD-L1, programmed cell death ligand 1.

Considering all these factors, treatment against HNSCCs has drawn great attention, and there have been few clinical trials in progress. The five main strategies to target CAFs in cancer can be summarized as follows (Fig. 6): (a) Halt or reverse CAF activation status: CAFs are always considered to be more pro-tumorigenic than normal or quiescent fibroblasts, and several drugs have been verified to be capable of inducing a NF-like phenotype [99]. (b) CAF depletion: FAP serves as a relatively specific marker for CAFs, either antibodies or immune treatments can be used to delete FAP-expressing cells [203]. (c) ECM targeted therapy: CAFs are the main producers of the ECM, which provides essential structural support for tumour cell growth and metastasis [204]. (d) Block the effect of secreted molecules from CAFs: Blocking the effect of CAF-secreted cytokines or relevant downstream pathways is considered an efficient and controllable strategy to tackle the effect of CAFs [205, 206]. (e) Combined treatment with other therapies: One of the main impacts of CAFs on cancer cells is to induce therapy resistance, and some treatments were developed to tackle this effect by either blocking resistance-related molecules or shared pathways by CAFs and tumour cells [55, 115].

## Conclusions, challenges, and perspectives

In the present review, we describe several CAF-mediated signalling pathways which induce a supportive role in cancer progression and treatment resistance. The bi-directional interactions between CAFs and HNSCC cells within the TME influence the growth and secretion profile of both cellular components.

Currently, various therapeutic strategies are being developed with CAFs or their functional mediators as direct targets. Moreover, many anticancer agents previously tested in humans may also target CAFs or their precursors, as CAFs share multiple common pathways with HNSCC cells. Fibroblasts are close partners of cancer cells and can function as either positive or negative regulators of tumour growth [33, 188]. Usually, quiescent fibroblasts and tumour-restraining CAFs were more present in early-stage cancers, while tumour promoting CAFs were frequently detected in advance staged HNSCCs. [207]. The underlying mechanisms that balance the tumour promotion or tumour inhibition effects of CAFs are mostly unknown, and the outcomes of some anti-CAF treatments were not as expected. [208]. Non-selective FAP-targeting drugs [209], tagged FAP-targeting drug [210], T cell-mediated CAFs depletion [203] and BMP pathway inhibitor [203] have been tested in vitro or in vivo, while only some of them showed promising results. The challenges and failures faced in direct CAF targeting approaches are a result of the lack of specific CAF markers and CAF heterogeneity. Thus, further research is required to identify pro-tumourigenic CAFs to improve therapeutic efficacy.

On the other hand, key signalling pathway components and CAF-derived factors were predicted or found to have great potential for targeted therapy. Since the crosstalk between cancer cells and CAFs is mediated by complex signalling networks, we propose a therapeutic approach, that is to target effector HNSCC cells activated by CAFs via blocking the effect of CAF-secreted molecules. TGF-β can be a direct therapeutic target, as TGF-β receptor III is found as a tumour-CAFs shared target in OSCC. Simultaneous perturbation of TβRIII in OSCC cells and their adjacent CAFs effectively inhibits tumour growth in vivo and shows superiority to the effect in either cell type alone [211]. CAFs secreted HGF-activated c-Met pathway has been linked to cetuximab resistance in HNSCC, and co-targeting both receptors has demonstrated inhanced ability to sensitize cells to EGFR-targeted therapies [102]. Met tyrosine kinase inhibitor crizotinib [175], and anti-HGF mAb-cetuximab dual treatment for advanced pan refractory HNSCC [177] has shown preliminary effect on HNSCC control. The clinically used IL-6R antibody Tocilizumab has shown promise in combination with standard anticancer treatments in ovarian [212], breast [205] and melanoma cancers [213]. However, clinical trials in HNSCC have so far been inconclusive. As a key modulator of immune environment in HNSCCs, CAFs have been implicated as potential mediators of checkpoint immunotherapy response. Different CAFs subtypes were identified in HNSCCs that can be used as a biomarker of response and resistance in immune checkpoint inhibitors [26]. It is now clear that HPV+ HNSCCs are more responsive to immune checkpoint inhibitors, than HPV- tumours, and CAFs were considered to play an important role in this differential response [110, 214].

Collectively, CAF-mediated signalling pathways and the crosstalk between CAFs and HNSCC influence tumour progression and therapy response and should be considered as important targets for personalised treatment of HNSCC and other cancer types.

## Data Availability

All data included in this review are available upon request by contact with the corresponding author.
